# *Thaumatin-Like Protein* (*TLP*) Gene Family in Barley: Genome-Wide Exploration and Expression Analysis during Germination

**DOI:** 10.3390/genes11091080

**Published:** 2020-09-16

**Authors:** Irfan Iqbal, Rajiv Kumar Tripathi, Olivia Wilkins, Jaswinder Singh

**Affiliations:** Plant Science Department, McGill University, 21111 Lakeshore Rd., Quebec, QC H9X3V9, Canada; Irfan.iqbal@mail.mcgill.ca (I.I.); rajiv.tripathi@umanitoba.ca (R.K.T.); Olivia.wilkins@umanitoba.ca (O.W.)

**Keywords:** barley, cereals, thaumatin like-proteins, phylogenetics, expression analysis

## Abstract

Thaumatin-like Proteins (TLPs) are known to play a vital role in plant defense, developmental processes and seed germination. We identified 19 *TLP* genes from the reference genome of barley and 37, 28 and 35 *TLP* genes from rice, *Brachypodium* and sorghum genomes, respectively. Comparative phylogenetic analysis classified the *TLP* family into nine groups. Localized gene duplications with diverse exon/intron structures contributed to the expansion of the *TLP* gene family in cereals. Most of the barley *TLPs* were localized on chromosome 5H. The spatiotemporal expression pattern of *HvTLP* genes indicated their predominant expression in the embryo, developing grains, root and shoot tissues. Differential expression of *HvTLP14*, *HvTLP17* and *HvTLP18* in the malting variety (Morex) over 16–96 h of grain germination revealed their possible role in malting. This study provides a description of the *TLP* gene family in barley and their possible involvement in seed germination and the malting process.

## 1. Introduction

*Thaumatin-like proteins* (*TLPs*) are part of a large pathogenesis-related (PR) gene family, involved in a broad range of defense and developmental processes in plants, fungi and animals [1]. In plants, *TLPs* are members of the PR-5 gene family including permatin, osmotin and osmotin-like proteins (OLPs), with their synthesis mainly triggered in response to biotic and abiotic stress. However, their expression is also developmentally regulated during seed germination [2] and fruit ripening [3], when they perform defense and development-related functions [4].

*TLPs* have high sequence similarity with the sweet-tasting disulfide thaumatin protein, which was initially identified in the West African shrub *Thaumatococcus daniellii* [5]. *TLPs* are highly conserved 24–34 kDa proteins with 225–319 amino acid residues [6]. They bear five to eight disulfide linkages, depending upon their number of cysteine residues, which range from 10 to 16. These disulfide structures provide stability and resistance to pH, high-temperature-induced denaturation and protease degradation [7]. *TLPs* with 10 conserved cysteines are designated as small *TLPs* and were identified in various monocotyledonous and coniferous plant species [6,8,9].

*TLPs* are involved in plant defense against numerous biotic and abiotic stresses [6]. Overexpression of *TLPs* results in induced resistance against downy mildew, *Macrophomina phaseolina*, *Phytopthora infestans* and salinity stress [10,11,12]. *TLPs* have roles in plant development and physiology, including tolerance to freezing stress [13], antifungal activity [12], fruit ripening [14] and seed germination [15]. *TLPs* were identified in various, plants including *Arabidopsis*, barley, black cottonwood, maize, moss and rice [2,6,16].

Recently, the role of *TLPs* was demonstrated in germinating barley seeds during the malting process [2]. Barley grain endosperm is composed of 75% non-starch polysaccharides, such as (1, 3, 1, 4)-β-D glucan “(hereafter, β-glucan)” and arabinoxylans [17]. A lower β-glucan content is considered as a desirable trait in determining a grain’s malting quality [18]. A barley TLP (HvTLP8), bearing a carbohydrate-binding motif (CQTGDCGG), was implicated in the redox-regulated interaction with β-glucan [2]. In addition, barley *TLPs* were found to be involved in antifungal activity [19], antimicrobial activity [20] and carbohydrate binding [21].

Considering the importance of *TLP* genes associated with various defense, development and physiological responses, as well as the diversity of *TLP* gene members in different plant species, it is critical to investigate the global status and evolution of the *TLP* gene family in barley and other cereals employed in the brewing industry, especially sorghum (a cereal used to produce gluten-free beer). As little information exists regarding the status of *TLP* gene family members in cereal grains, we were particularly interested in TLPs possessing a carbohydrate-binding domain that may interact with different polysaccharide moieties during the germination and malting processes. Our data assess the global status of the *TLP* gene family and expand our knowledge of its instances in rice, *Brachypodium*, sorghum and barley. The availability of updated genome sequence databases of several plant species facilitates our exploration of the *TLP* gene family’s status in cereals.

## 2. Materials and Methods

### 2.1. Sequence Retrieval and Identification of Thaumatin-Like Proteins in Cereals

**Figure 1:**
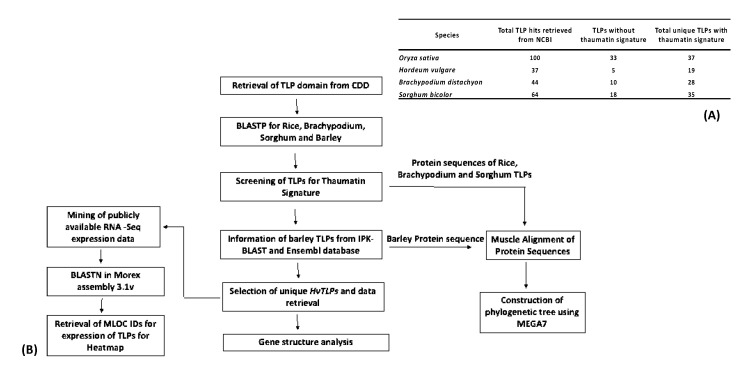
(**A**) Number of *thaumatin-like protein* (*TLP*) genes in rice, barley, *Brachypodium* and sorghum. (**B**) Pipeline for the bioinformatics analysis. MLOC ID: barley gene ID/ transcript; CDD: Conserved Domain Database; BLASTP: protein blasting; TLPs: Thaumatin-like Proteins.

The TLP domain sequence was retrieved from a Conserved Domain Database (CDD) (https://www.ncbi.nlm.nih.gov/cdd) and used as a query sequence for protein blasting (BLASTp) (https://blast.ncbi.nlm.nih.gov/Blast.cgi) in National Center for Biotechnology Information NCBI (https://www.ncbi.nlm.nih.gov/) database. The BLASTp was performed using a nonredundant protein sequence database using an *e* value cutoff of *e*^−10^ to retrieve TLP protein sequences for barley, rice, sorghum and *Brachypodium*. Only the longest gene models were selected for further analysis. In addition, identified barley *(Hordeum vulgare*) *HvTLP* gene sequences were also verified using IPK barley (https://webblast.ipk-gatersleben.de/barley_ibsc/) and the Ensembl Plants database (http://plants.ensembl.org/Hordeum_vulgare/Info/Index) in an effort to retrieve gene IDs. Further screening for analysis was performed with only those genes selected with the thaumatin family signature. The pipeline for the bioinformatic analysis is illustrated in Figure 1B. Identified genes were also confirmed as TLPs from the Simple Modular Architecture Research Tool (SMART) (http://smart.embl-heidelberg.de/) and Pfam (https://pfam.xfam.org/) databases.

### 2.2. Characteristics of TLPs and Phylogenetic Analysis

The characteristics of HvTLPs, including isoelectric point (pI) and molecular weight (MW), were predicted using ExPASy ProtParam (https://web.expasy.org/protparam/) [22]. The subcellular location was projected using Softberry, a web-based tool (http://www.softberry.com/berry.phtml). The transmembrane (TM) regions and signal peptides predictions were made through TMHMM 2.0 (http://www.cbs.dtu.dk/services/TMHMM-2.0/) and SignalP 5.0 (http://www.cbs.dtu.dk/services/SignalP/), respectively [23,24]. Multiple sequence alignment of amino acid sequences of *TLPs* from rice, sorghum, barley and *Brachypodium* (Table S1) served to construct a phylogenetic tree. Multiple sequence alignments were performed using the Multiple Sequence Comparison by Log-Expectation (MUSCLE) (https://www.ebi.ac.uk/Tools/msa/muscle/) alignment tool operated with default settings [25]. An unrooted Neighbor-Joining phylogenetic tree was generated using the MEGA v7 program (1000 bootstrap replicates) [26,27,28,29].

### 2.3. Chromosomal Location, Exon/Intron Structure and Alternative Splice Variants Analysis of Barley TLP Genes

Chromosomal location, intron/exon structures and predicted alternative splice variants of the *HvTLPs* were determined using the barley Ensembl database (http://plants.ensembl.org/Hordeum_vulgare/Info/Index). Initially, 32 TLP proteins with the thaumatin signature were identified in Genebank using BLASTp, as previously described; upon blasting these sequences against the Ensembl database, 32 protein sequences were determined to be aligned to only 19 genetic loci. In all subsequent analyses, only the longest gene isoforms were used. These are provided in Table S2. Exon/intron structures of the barley *HvTLP* genes were illustrated by using the online Gene Structure Display Server (GSDS; http://gsds.cbi.pku.edu.cn), which displayed the gene length and exon position [30,31].

### 2.4. Gene Expression Analysis of Novel Barley TLPs in Different Tissues of a Barley Variety, Morex

We used the publicly available Morex RNA-seq database (International Barley Genome Sequencing 2012) to study the expression patterns of 19 *HvTLP* genes, including 11 novel *TLPs* active in eight different developmental stages, tissues and inflorescence treatments. Caryopses (CAR5) expression data were collected from developing grains with bracts removed (5 Days post anthesis (DPA), while roots (ROO) were collected from seedling roots (0.10 m shoot stage), Shoot (LEA) was taken from seedling shoots (0.10 m shoot stage), embryonic tissue (coleoptile, mesocotyl and seminal roots) (EMB) was collected from 4-day embryos dissected from germinating grains, CAR15 was collected from developing grain, bracts were removed (15 DPA), inflorescences (INF1) was taken from young, developing inflorescences (5 mm), INF2 was taken from developing inflorescences (1–1.5 cm) and internode (NOD) was taken from developing tillers at the 6-leaf stage and the third internode. A heat map of *HvTLP* transcript abundance was generated by using the online Mev tool (http://mev.tm4.org/) with the average hierarchical clustering method. 

### 2.5. Plant Material and Growth Conditions

Seeds of two barley varieties (Malting: Morex and Feed: Steptoe) were obtained from Plant Gene Resources of Canada (PGRC) and used as the experimental material. Mature barley seeds were surface sterilized with 20% bleach and rinsed with distilled water three times. They were germinated in the dark on wet filter paper in sterile petri dishes. Germinating seeds were collected at 16, 48 and 96 h (Figure S1) and were flash-frozen in liquid nitrogen and stored at −80 °C until further processing. 

### 2.6. Total RNA Isolation and cDNA Synthesis

Total RNA was extracted from the germinating seed tissue using a Spectrum Plant Total RNA kit, following the manufacturer’s instructions (Sigma-Aldrich, St. Louis, MO, USA). Quantification of RNA concentration for all samples was performed using NanoDrop ND-1000 (NanoDrop Technologies, Wilmington, DE, USA), and samples were electrophoresed on 1.2% agarose to test RNA quality and integrity. To remove the genomic DNA contamination, each sample was treated with DNase I (Invitrogen, Carlsbad, CA, USA). The samples were incubated at 23 °C for 15 min. Then, 1 μL of 25 mM Ethylenediamine tetra acetic acid (EDTA) was added to each sample, followed by incubation at 65 °C for 10 min to terminate the reaction. cDNA was synthesized using an AffinityScript qPCR cDNA synthesis kit from 500 ng of total RNA, according to the manufacturer’s instructions (Agilent Technologies, West Cedar Creek, Texas, USA). 

### 2.7. RT-PCR Analysis

Primers were designed for *HvTLPs* using the Integrated DNA Technologies (IDT) primer quest tool (https://www.idtdna.com/PrimerQuest/Home/Index) (additional file 3; Table S3). Transcript abundance was determined with the use of RT-PCR analysis using GoTaq^®^ G2 Green Master Mix. A reaction volume of 20 μL, containing 1 μL of cDNA, was used for each sample. The amplification conditions were 95 °C for 2 min, followed by 30 cycles at 95 °C for 30 s (annealing temperature was adjusted according to the primers provided in (Table S3), 72 °C for 30 s and final extension at 72 °C for 5 min. *β-Actin* and *GAPDH* were used as expression controls [32]. Quantification of gel band intensities were performed using the GelAnalyzer 19.1 software (http://www.gelanalyzer.com/). Expression levels in each sample were calculated relative to *HvActin*.

### 2.8. Statistical Analysis

Data were analyzed using IBM Statistics SPSS Version 24 (SPSS Inc., Chicago, IL, USA). Comparison of the means was performed with an independent Student’s *t*-test at the significance level of 0.05.

## 3. Results

### 3.1. Identification of TLP Gene Family in Cereals

A total of 19, 28, 35 and 37 *TLP* genes were identified in barley, *Brachypodium*, sorghum and rice, respectively (Figure 1A). The TLP domain was retrieved from CDD and used as a query to perform BLASTp in NCBI to identify *TLP* genes in barley, rice, sorghum and *Brachypodium*. Protein sequences of *TLP* candidate genes were confirmed by Pfam (Domain number: PF00314) and SMART for the presence of the thaumatin family signature G-x-[GF]-x-C-x-T-[GA]-D-C-x-(1,2)-[GQ]-x-(2,3)-C. Out of the 19 genes identified in barley, eight were previously reported (*HvTLP1-8*) [2]. The 11 new coding sequences of *HvTLP* genes ranged from 522 to 1080 base pairs, found to be localized on chromosomes 1H, 3H, 4H, 5H and 7H.

### 3.2. HvTLP Protein Feature Analysis

Barley TLP protein length varied from 173 to 359 amino acid residues, with cysteine residues ranging from 10 (HvTLP1 and HvTLP2) to 24 (HvTLP17) (Table 1 and Figure S2). In addition to previously known HvTLP1, HvTLP2 and HvTLP8, only one new TLP, HvTLP17, contained the carbohydrate binding CQTGDCGG motif in the amino acid sequences. A similar carbohydrate-binding motif (CQTGDCQG) was found in HvTLP14, except for the substitution of glycine (G) to glutamine (Q). Subcellular localization predictions indicated that most of the *TLPs* were located in the extracellular regions, except HvTLP14 and HvTLP17, which localized in the plasma membrane region (Table 1). Results from transmembrane (TM) domain analysis showed that 12 out of 19 TLPs contained one TM region, while HvTLP14 possessed two TM domains (Table 1). Signal peptide analysis revealed that all HvTLPs contained an N-terminal signal peptide (Table 1), suggesting the likelihood of being localized in the extracellular space [33].

### 3.3. Phylogenetic Relationship of TLP Genes in Barley, Rice, Brachypodium and Sorghum

**Figure 2:**
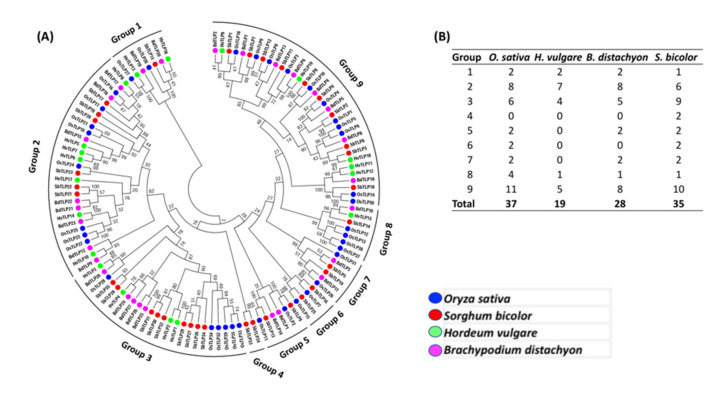
(**A**) Phylogenetic analysis of TLP protein family among cereals. The TLP domain was identified and the phylogenetic tree was constructed using a neighbor-joining method with 1000 bootstraps. Terminal tree markers are indicative of species (barley, bright green; rice, blue; sorghum, red; *Brachypodium*, pink). (**B**) Group wise distribution of cereal TLPs in the phylogenetic tree.

A phylogenetic tree was constructed using the Neighbor-Joining method based on 119 TLP protein sequences from sorghum, rice, barley and *Brachypodium* (Figure 2A). These protein sequences were arranged into nine groups. Barley contained only 19 *TLPs*, fewer than the other species, however, this contrasted with one previous study [2] where only eight *TLPs* were reported in barley. The number of TLPs in rice were twice those in barley (Figure 2B). A maximum number of (34) TLPs from the four-plant species were clustered in group 9, whereas only six TLPs were found in groups 5 and 7. Interestingly, seven (HvTLP5, 6, 7, 8, 10, 14 and 17) out of 19 TLPs were found in group 2. These data suggested that orthologues for *HvTLP13* and *HvTLP4* genes are missing in sorghum and *Brachypodium*, respectively. Barley TLPs in group 2 exhibited 35 additional amino acids residues at the N-terminal end of the TLP domain. Corresponding genes of *HvTLPs* in group 3 possessed a single exon, except *HvTLP1*, which exhibited two exons.

In general, barley TLPs showed a closer relationship with *Brachypodium,* followed by rice and sorghum. Interestingly, some *HvTLPs* located adjacent to each other on chromosome 5H were found to be clustered in the same group (group 2), i.e., HvTLP5, 6, 7 and HvTLP12, 18 (Figure 2). Similarly, phylogenetic analysis revealed that some members of rice and sorghum *TLPs* were clustered together, especially in group 3.

### 3.4. Gene Structure Analysis and Identification of Thaumatin Signature in Barley TLPs

**Figure 3:**
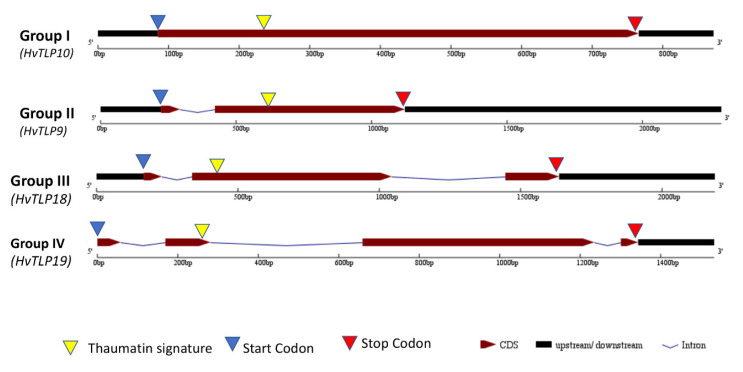
Structural features of barley *TLP* genes. From left to right is the 5′ to 3′ region containing untranslated region (UTR) in black, with maroon representing exons, blue triangles representing the start codons, red triangles for the stop codons, black narrow lines with slits symbolizing an intron and yellow triangles representing the thaumatin signature.

Classification of 19 *TLPs* from barley based on the number of exons (ranging from 1 to 4) resulted in the formation of four (I–IV) groups (Figure 3). Eleven *TLPs* (*HvTLP2*, *3*, *4*, *6*, *8*, *10*, *11*, *12*, *13* and *14*) with one exon were classified into Group I, while Group II included six *HvTLPs* (*HvTLP1*, *5*, *6*, *9*, *16*, *17* and *18*). However, Group III (*HvTLP18*) and Group IV (*HvTLP19*) possessed one *TLP* gene member each. Except for Group I, all members of the other groups contained nucleotides encoding for the thaumatin family signature G-x-[GF]-x-C-x-T-[GA]-D-C-x-(1,2)-[GQ]-x-(2,3)-C in their second exon. The thaumatin family signature was manually searched in the protein sequences of the 37 genes, but only 19 unique genes with the complete thaumatin signature were selected.

### 3.5. Alternative Splicing in HvTLPs

Alternative transcripts produced protein isoforms with amino acid sequence differences ranging from modified cellular properties to loss of protein function. The barley genome from the Ensembl database was mined to identify any alternative splicing event for *HvTLP* genes. Predicted alternative splice variants of 15 *HvTLPs* were identified, contributing 83% toward the total *HvTLP* splice variants. The maximum number of splice variants for a gene were 14 (*HvTLP17*); four genes *(HvTLP2*, *HvTLP3*, *HvTLP10* and *HvTLP13)* showed no splice variants (Table S4).

### 3.6. Spatiotemporal Expression Pattern of HvTLPs

**Figure 4:**
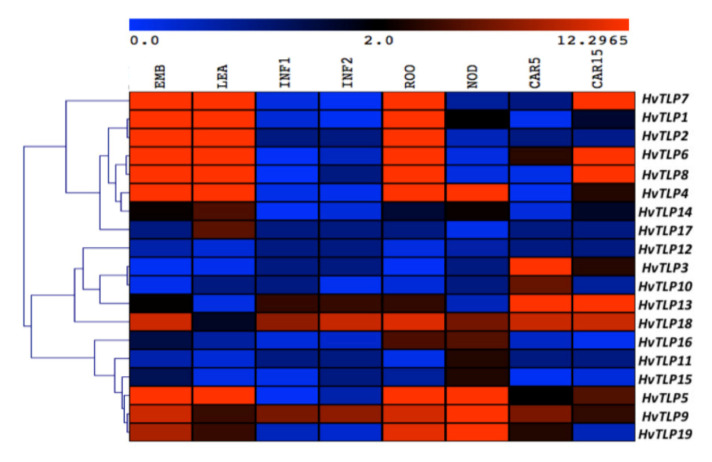
Spatiotemporal expression of *HvTLPs* genes in different barley tissues. The color scale bar at the top of heat map represents FPKM (Fragments per kilobase of exon model per million reads mapped) normalized log2-transformed values based on “Morex” RNA-seq data, representing high and low expression, respectively. Embry (EMB), shoot (LEA), inflorescences (INF1, INF2), root (ROO), tillers (NOD) and developing grains (CAR5 and CAR15) tissues were used for expression profiling. Details regarding these tissues are explained in Section 2.

RNA-sequencing (RNA-seq) data provides important information for predicting the function of genes. To understand the developmental role of previously known and newly identified novel barley *TLP* genes, we performed a comprehensive expression analysis using barley gene ID/ transcript (MLOC ID) for the *TLPs* (Table S5) from the Morex Genes-Barley RNA-seq database (https://ics.hutton.ac.uk/morexGenes) to investigate their spatiotemporal expression pattern. Based on the data shown in the extracted heatmap (Figure 4), *HvTLPs* exhibited differential expression in developing grains (CAR5 and CAR15), root (ROO), shoot (LEA), embryo (EMB), young inflorescence (INF1), developing inflorescence (INF2) and tillers (NOD). The *HvTLP13* and *18* genes were found to be expressed to a greater extent in CAR5 and CAR15, indicating the possibility of these genes being involved in the development of these tissues. Only *HvTLP9*, *5* and *19* showed higher expression profiles in roots and tillers, suggesting that they might be involved in the development of these tissues, but they were not expressed in INF1 and INF2, except *HvTLP9*. *HvTLP1*, *2*, *4*, *5*, *6*, *7*, *8*, *9*, *18* and *19* exhibited elevated expression levels in EMB, suggesting their possible role in embryo development within the barley grain. 

### 3.7. Transcript Abundance of HvTLPs during Different Stages of Barley Seed Germination

**Figure 5:**
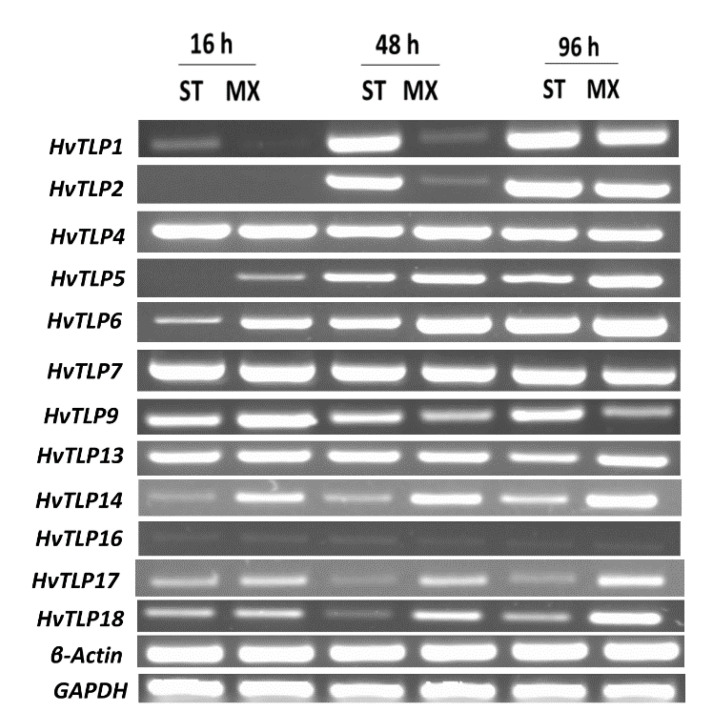
Transcript abundance of *HvTLP* genes at different stages of seed germination. Gel representation of the expression profiles of *HvTLPs* (*1*, *2*, *4*, *5*, *6*, *7*, *9*, *13*, *14*, *16*, *17* and *18*) and the housekeeping genes *β-actin* and *GAPDH* at 16 h, 48 h and 96 h of germination in malting (MX = Morex) and feed (ST = Steptoe) varieties of barley using RT-PCR. 1.2% agarose gel was used to resolve the amplified bands.

The transcript abundance of 16 *TLPs* was measured through RT-PCR using two housekeeping genes, *β-ACTIN* and *GAPDH*, as controls (Figure 5). The *HvTLP4* and *HvTLP7* genes exhibited greater expression at all time intervals, but with no difference between the malt and feed varieties. Similarly, *HvTLP13* expression constantly increased across all stages of seed germination. However, its expression was reduced in Steptoe (feed variety) at 96 h of germination. Transcripts of *HvTLP5* and *HvTLP6* accumulated continuously as germination proceeded from 16 through 96 h in Morex (malt variety). No or low expression of *HvTLP5* and *HvTLP6* was observed in Steptoe (feed variety) at 16 h. The transcript abundance levels of *HvTLP1* and *HvTLP2* were higher in Steptoe at 48 h of germination. Expression of *HvTLP9* was higher in Morex at 16 h of imbibition but reduced dramatically thereafter. Higher transcript abundance of *HvTLP14*, *HvTLP17* and *HvTLP18* was observed in Morex compared to Steptoe at all time points of the germination process. No gene expression was observed for *HvTLP3*, *HvTLP10*, *HvTLP12* or *HvTLP15* in either Steptoe or Morex at any stage of germination between 16 and 96 h of imbibition (Figure 5 and Figure S3).

## 4. Discussion

Availability of whole-genome sequences of rice, sorghum, *Brachypodium* and barley allowed for genome-wide exploration of the *TLP* gene family among these crops (Figure 1B). Here, the overall status of *TLP* genes in rice, sorghum, *Brachypodium* and barley are reported, i.e., 37, 35, 28 and 19 genes, respectively (Figure 1B). It is worth noting that in barley, only eight *TLP* genes were previously reported [2]. As our previous work established that *HvTLP8*, a *TLP*, was differentially expressed during germination in malt and feed varieties and played an important role in sequestering β-glucan during the malting process [2].The major focus of the present study was to perform a genome-wide exploration of *TLPs* in barley, especially during germination. Barley grains contain the non-starch polysaccharide β-glucan, and its higher quantity in the grain affects the brewing process [34]. The amino acid sequence of *HvTLP*8 gene possesses a carbohydrate-binding domain and the binding motif CQTGDCGG, allowing it to bind to β-glucan in a redox-dependent manner [2]. We identified two further barley *TLP* genes that also contained the binding motif, indicative of their interaction with carbohydrate moieties, which requires further investigation. Previously, 44 *TLP* genes were reported in rice [16]. However, our careful analysis indicated that, based on the presence of the thaumatin family signature, the rice genome contains only 37 true *TLP* genes. 

**Figure 6:**
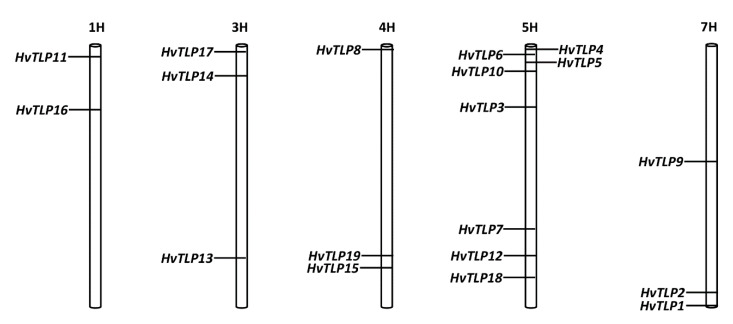
Map locations of *HvTLP* genes in barley genomes. Exact positions are shown in Table 1.

Furthermore, we identified 11 new *TLPs* in barley. Previously, the eight-known barley *TLPs* were classified into two groups (Group 1 and 2) based on the number of cysteine residues (10 and 16) and were localized on chromosomes 4H, 5H and 7H only [2]. However, the present data emphasize that *TLP* genes can also be assigned to chromosomes 1H and 3H (Figure 6). Generally, TLPs are considered cysteine-rich proteins with a maximum number of 16 cysteine residues, however, our study revealed the presence of a greater number of cysteine residues in some TLPs. For example, HvTLP17 contains 24 cysteine residues (Table 1). It is well documented that cysteine residues result in the formation of disulfide linkages, which provides protein stability, especially when exposed to extreme pH, temperature and protease degradation conditions, etc. [7]. Plant TLPs are documented as proteins ranging from 21 kDa to 26 kDa in size. However, the molecular weights of the new TLPs identified in the present study were calculated to be as high as 41 kDa. Moreover, *TLP* genes were identified in a wide range of plants from mosses to wheat, bearing a complex hexaploid genome [6,35] and suggesting that this gene family expanded throughout different plant species during the process of evolution. To better understand the diversification of this gene family in small grain cereals, we performed a phylogenetic analysis of predicted TLP protein sequences from rice, sorghum, *Brachypodium* and barley (Figure 2A). A total of 119 TLP proteins from four different plant species were classified into nine groups. A maximum number (34) of TLP proteins were clustered in group nine (Figure 2B). Previously, 44 *TLP* genes were reported in rice [16], which were identified by keyword searches in the rice genome, some of which did not possess the thaumatin family signature. However, by using our gene identification approach (see Section 2), 37 and 35 true *TLP* genes bearing the thaumatin family signature were found in rice and sorghum, respectively. Both rice and sorghum genomes share 94% of high-confidence genes [36], potentially reflecting similarity in the number of *TLP* genes between rice and sorghum. Rice and sorghum genomes possess nearly twice as many *TLP* genes (35–37 genes) compared to barley’s 19 *TLPs.* This is probably due to segmental and whole genome duplication events in rice [37,38]. However, it appears from our data that the expansion in rice, barley, *Brachypodium* and sorghum was probably due to localized gene duplications, since many small TLP groups, located in close proximity on the same chromosome, demonstrate high sequence similarity. This situation could be clearly observed for the *TLPs* located on chromosome 5H (Figure 6). Therefore, localized gene duplication events may be the primary reason for *TLP* gene family expansion in barley.

Intron structures are also very important in determining the complexity of the genetic structures of eukaryotic organisms [39]. Notably, 10 *HvTLP* genes were found without introns, whereas the remaining *HvTLP* genes contained at least one intron (Figure 3), suggesting that variation in the number of intron/exons among *HvTLP* genes might play an important role in controlling their function during the growth and development of barley. Ambient temperature causes alternative splicing that functions as a molecular thermometer in plants. Recently, alternative splicing in *SQUAMOSA promoter-binding protein-like* (*SPL*) genes in barley [40] was identified, with differential levels of accumulation during the vegetative to reproductive phase transition. Alternative splicing is also involved in the process of seed germination in barley [41]. Alternative splicing in *FT* genes was identified as a mechanism of flowering regulation in *Brachypodium* [42]. *ARF8.4*, a splice variant of *AUXIN RESPONSE FACTOR 8*, is involved in stamen development in *Arabidopsis* [43]. Likewise, we also investigated alternative splicing events in *HvTLP* genes and found that about 83% of *HvTLP* genes produce splice variants, implying their possible diverse roles in barley growth and development (Table S4).

Gene expression also provides a clue to the possible functions of genes in the absence of mutation. Therefore, we examined the spatiotemporal expression patterns of *HvTLPs* in eight different barley tissues (Figure 4). The heatmap-based transcript profiles of *HvTLPs* showed that expression was differential throughout different tissues; however, high expression levels of (*HvTLP1*, *2*, *4*, *5*, *6*, *7*, *8*, *9*, *18* and *19*) were found in EMBs (Figure 4), showing that they might have possible roles in embryo development during germination.

Seed germination and seedling development are important stages of plant development. Barley grain germination is a key step in the process of malting, which is highly stage-specific for efficient brewing. Differential gene expression was previously observed after 18 h of seed germination [44]. Our data showed that most of the *HvTLPs* exhibited higher expression levels between 48 to 96 h in the germinating grains. *HvTLP4* and *HvTLP7* showed elevated levels of expression in all stages of germination, regardless of malt or feed varieties, suggesting their possible role during seed germination. However, at 16 h of germination, *HvTLP5* and *HvTLP6* expression levels were found to be higher in Morex when compared to Steptoe, consistent with the recent expression analysis of *HvTLP8*, where higher transcript abundance at 16–96 h seed germination was observed in malting varieties compared to feed varieties [2]. We identified three additional genes (*HvTLP14*, *HvTLP17* and *HvTLP18*) which mimic the expression pattern of *HvTLP8* (Figure 5 and Figure S3). Whether these play a similar role in influencing the grain biochemistry during malting and brewing process requires further investigation. These *TLPs* could, however, be considered potential new candidates for the breeding of barley for malting and brewing. Previously, we identified other important genes involved in the germination, dormancy [45] and malting processes [2]. As no data for germinating grains are currently available in the publicly accessible Morex RNA-seq database, we performed validation of *HvTLP* gene expression by measuring transcript abundance in germinating barley grains of malt and feed varieties at different stages. The motivation to conduct these experiments is derived from our recent data, where differential expression of *HvTLP8* was associated with β-glucan levels in germinating barley grains [2]. Reduction of β-glucan is an important breeding objective in barley malt varieties. The association of *TLPs* with β-glucan could also lead to the development of high-value and high-fiber cereals by knocking out expression using new clustered regularly interspaced short palindromic repeats-based approaches.

## 5. Conclusions

Our results provide novel information about the status of the *TLP* gene family in cereals, as knowledge about this gene family is scarce. Due to the availability of sequencing data and new tools, we were able to identify new *TLPs* which were previously unknown. Interestingly, some of these TLPs possess a greater number of cysteine residues than previously thought. One cysteine rich TLP, HvTLP8, was found to be associated with β-glucan, with the interaction was found to be dependent on the redox status. Therefore, the role of newly identified, cysteine rich TLPs demands further elucidation, especially in terms of activity and stability. It is worth noting that some *TLPs* expressed during germination and their polymorphisms in malt and feed varieties could help in the understanding of their roles in malting and in the development of novel molecular markers for breeding of the future generation of malting barley varieties. New information regarding the *TLP* gene family in cereals could also be helpful in further investigation of possible functional responses toward development, physiology and defense-related stimuli in cereals.

## Figures and Tables

**Table 1:** Characteristics of newly identified barley *TLP* genes.

			Deduced Protein									
Gene Name	Gene Symbol	CDS Length (bp)	a.a	p*I*	MW (kDa)	No. Cysteine Residues	Chrom-osomal Location	Genomic Location	Exon No.	Subcellular Localization	Signal Peptide	TM Domain
*HvTLP1*	HORVU7Hr1G122120.1	522	173	4.33	17.548	10	7H	655448674-655449693	2	Extracellular	N-terminal	1
*HvTLP2*	HORVU7Hr1G122100.1	522	173	4.51	17.576	10	7H	655345402-655345932	1	Extracellular	N-terminal	1
*HvTLP3*	HORVU5Hr1G017530.1	528	175	5.02	18.048	10	5H	67689233-67690149	1	Extracellular	N-terminal	1
*HvTLP4*	HORVU5Hr1G005290.1	690	229	6.45	24.325	10	5H	8764295-8765150	1	Extracellular	N-terminal	1
*HvTLP5*	HORVU5Hr1G005180.4	744	247	6.48	26.062	16	5H	8598500-8736900	2	Extracellular	N-terminal	1
*HvTLP6*	HORVU5Hr1G005180.6	681	226	7.33	23.725	16	5H	8614116-8615298	1	Extracellular	N-terminal	1
*HvTLP7*	HORVU5Hr1G051970.4	738	245	8.13	25.61	16	5H	406435574-406436533	2	Extracellular	N-terminal	0
*HvTLP8*	HORVU4Hr1G002650.2	768	255	8.11	26.821	16	4H	5073727-5074962	1	Extracellular	N-terminal	1
*HvTLP9*	HORVU7Hr1G057350.1	771	256	4.83	26.959	16	7H	248978165-248980455	2	Extracellular	N-terminal	1
*HvTLP10*	HORVU5Hr1G005190.1	681	226	5.57	23.177	16	5H	8603703-8604574	1	Extracellular	N-terminal	0
*HvTLP11*	HORVU1Hr1G005760.1	786	261	5.18	26.106	17	1H	12508776-12510365	1	Extracellular	N-terminal	0
*HvTLP12*	HORVU5Hr1G084650.2	840	279	6.83	27.804	18	5H	573025822-573026929	1	Extracellular	N-terminal	1
*HvTLP13*	HORVU3Hr1G086510.1	753	250	7.47	26.017	17	3H	617523511-617524882	1	Extracellular	N-terminal	0
*HvTLP14*	HORVU3Hr1G012100.9	1026	341	5.33	38.477	22	3H	26305708-26310551	1	Plasma membrane	N-terminal	2
*HvTLP15*	HORVU4Hr1G066130.4	780	259	4.79	25.744	16	4H	550925586-550926443	2	Extracellular	N-terminal	0
*HvTLP16*	HORVU1Hr1G021650.3	777	258	8.43	27.097	17	1H	88073476-88075764	2	Extracellular	N-terminal	1
*HvTLP17*	HORVU3Hr1G002210.5	1080	359	7.72	40.844	24	3H	4627368-4637925	2	Plasma membrane	N-terminal	1
*HvTLP18*	HORVU5Hr1G112160.1	960	319	4.85	31.991	16	5H	638398994-638401180	3	Extracellular	N-terminal	0
*HvTLP19*	HORVU4Hr1G063730.7	789	262	6.07	27.062	19	4H	533492849-533494381	4	Extracellular	N-terminal	0

## Supplementary Materials

The following are available online at https://www.mdpi.com/2073-4425/11/9/1080/s1: Table S1: Amino acid sequences of barley, rice, sorghum and *Brachypodium* *TLPs* used in this study; Table S2: Information regarding unique genetic loci for barley *TLPs*; Table S3: List of primers used for expression analysis of *HvTLPs* in malt and feed varieties of barley at 16 h, 48 h and 96 h of seed imbibition; Table S4: Information regarding *HvTLPs* splice variants; Table S5: Gene IDs from Morex RNA-seq Assembly; Figure S1: Grain germination in Steptoe and Morex at different growth stages; Figure S2: Amino acid sequence alignment of barley *HvTLPs*. The highlighted sequences indicate the novel TLP11-14, TLP16, TLP17 and TLP19 with >16 cysteine residues. Sequence alignment was performed by using the MUSCLE alignment tool; Figure S3: Transcript levels of *HvTLPs* in malt (Morex) and feed (Steptoe) varieties during different stages of seed germination. Error bars were obtained from two measurements. Asterisks (*) above the bars indicate significant (*p* < 0.05) differences in expression levels between malt and feed varieties.
