# Supplementary material for: Thaumatin-Like Protein (TLP) Gene Family in Barley: Genome-Wide Exploration and Expression Analysis during Germination
Source: Genes (Basel). 2020 Sep 16;11(9):1080. doi: 10.3390/genes11091080 (PMC7564728; doi:10.3390/genes11091080)
Supplement: Supplementary file 1 [file genes-11-01080-s001.zip › Figure S2.pdf]

```
HvTLP3     --------------------------MARASPVVLVLVAAGLA-AG---ASATSTPLTITN
HvTLP13    --------------------------MGMASPKLLLLLATFLPSCGLLLADFAPMTLTVVN
HvTLP16    ------------------------MALLLPLFLSCILVLALPGPAV---LMVGGVTFHVTN
HvTLP11    ---------LTMAGGNGATTRRQLTLFPLHVTLVFQLLCAKR-------AAAATSFSFAN
HvTLP12    MTPIAAAGKKNNGAISTCSTCRQLIKLFPLHLIIVLLLGVAT--S---GGSAARSFSFHN
HvTLP15    ------------------------MEAARIASLLLLLGAVWSRAQPGADAAGTTVFTLRN
HvTLP18    ---------------------MATRAHRAPISLLVLLLASVT-------GVASKSFAITN
HvTLP9     -------------------MAQPSLLLGSPASIVLLLLSFFQ--G---PVAGAITFTFAN
HvTLP19    -----------------------MGIQRVCFVVALLCMCLRE--------GGAVTFTFLN
HvTLP1     ---------------------------MSTSAVLFLLLAVFA------AGASAATLNIKN
HvTLP2     ---------------------------MSTSAVLFLLLAVFA------AGASAATFNIKN
HvTLP4     -------------------------MAASSSALIILLAAALT------AGASARSFSITN
HvTLP10    ------------------------MASSSLSFILSLLLAIAA--------TDAITVHLLN
HvTLP5     ------------------------MAFSGVVHLIALVIAVAA------AATDATTITVVN
HvTLP6     -----------------------MASSRVVYLLAGLLLAALA------ATTDAATITVVN
HvTLP7     -----------------------MASSHVVSLLAGLLLAALA------ASTDAATITVVN
HvTLP8     MPFFLTTGTLKLYYVQGRGENTMASLPTSSVLLPILLLVLVA------ATADAATFTVIN
HvTLP14    ------------------MGTSSLSLHHPPPLLLLLRLLLLP------FVTAQTILNITN
HvTLP17    ------------------------MPASSALFLLLHLLLFLA------ATGEATTFSITN
                                                                  . . *

HvTLP3     RCSFTVWPAVAP-------AGLGTELHPGANWSVDESA-FDSPASIWGRTGCSFD-AAG-
HvTLP13    NCPYPVWPGIQGSAGSEVLEGGGFYLPSLSHRSFPAPA-HAWSGRIWARTGCTPA-GGE-
HvTLP16    KCPFPVWPAVAPNAGHPVLAAGGFFLPPGQSKRVGAPA--TWNGRFWGRTGCNFA-GTG-
HvTLP11    QCAHPVWVGALSGATSPQLARTGFYLAAGATSGLAAPSSGSWSGTFWARTGCAVDEGTG-
HvTLP12    ACAHPVWVGALNGATSPPLARTGFYLASGATDAIAAPSSGAWSGNFWARTGCAVD-SSTG
HvTLP15    NCTYTIWPATLSGNSAVAVGGGGFELAPGANVSFPAPT--GWSGRLWARTGCVAA-ASGA
HvTLP18    NCEYTVWPGILSSAGSPGMDSTGFALAPGESRTMPVPA--GWSGRLWGRTLCTTD-PAG-
HvTLP9     RCTDTVWPGLLSGSTSPPLETTGFALSPGQSRSLYGPT--GWSGRFWGRSGCNFN-G-G-
HvTLP19    RCTGTVWPGILSNAGTARIEPTGFALPPGAARALPFPT--GWSGRLWARTGCAQD-AAG-
HvTLP1     NCGSTIWPAGIP-------VGGGFELGSGQTSSINVPA-GTQAGRIWARTGCSFN-G-G-
HvTLP2     NCGSTIWPAGIP-------VGGGFELGSGQTSSINVPA-GTQAGRIWARTGCSFN-G-G-
HvTLP4     RCSFTVWPAATP-------VGGGRQLNGGETWNLDIPD-GTSSARIWGRTDCSFN-G-N-
HvTLP10    KCPYTVWPAAFP-------VGGGSRLDPGQAAAIQVPP-GTAGGRIWGRTGCNFD-ASG-
HvTLP5     RCSYTIWPGALP--------GGGARLDPGQSWQLNMPA-GTAGARVWPRTGCTFD-RSG-
HvTLP6     RCSYTVWPGALP--------GGGVRLDPGQSWALNMPA-GTAGARVWPRTGCTFD-GSG-
HvTLP7     RCSYTVWPGALP--------GGGVRLDPGQSWALNMPA-GTAGARVWPRTGCTFD-GSG-
HvTLP8     KCQYTVWAAAVP-------AGGGQKLDAGQTWSINVPA-GTTSGRVWARTGCSFD-GAG-
HvTLP14    RCPYTVWPAALP-------VGGGMRLDPGKTWILKVPY-DTTGGRVWARTGCSFD-GRG-
HvTLP17    GCPYTIWPAALP-------IGGGMQLDSGKQWTLNTGD--TNTTRLWARTGCSFD-GNG-
            *  .:* .             *  *       .           .* *: *

HvTLP3     SGLCRTADCG-SGLRCATTDPPAPVTRAQVA----SSEGFYHYGITTDKGFNLPLDL---
HvTLP13    QLRCATGDCG-GRLQCGGLGGAAPATLAQVSLH--HGNDQSSYGVSVVDGFNVGLSVTP-
HvTLP16    ---CLTGDCE-GRLACNGSIGAPPATLVEVSLHEDQSKGSS-YDVSVVDGYNLPVAVWT-
HvTLP11    RLACATADCGSGDVACDGRGPAPPVTLSEVTLAAPGSGGLDFYDVSLVDGFNLPVSVAP-
HvTLP12    RLACATADCGTGGVACAGRGPAPPVTLAEVTLAAPGGGGLDFYDASLVDGFNLPLSIAP-
HvTLP15    SLACATGDCG-GAVRCT-LGGAPPVTLAEFTLG--GADGKDFYDVSLVDGYNVGIGVAA-
HvTLP18    KFACVTGDCGSGRQDCAGGGAAPPATLAEFTMD--GNDGMDFYDVSLVDGYNLPMLVAPE
HvTLP9     KGTCVTGDCGSGEIECRGAGATPPATLAEFTLD--GDGGKDFYDVSLVDGYNLPMLVQP-
HvTLP19    RFACATGDCGTGTLECAGRDGATPATLAEFTLD--GGGHNDFYDVSLVDGYNLPILVEP-
HvTLP1     SGSCQTGDCG-GQLSCS-LSGQPPATLAEFTIG--GGSTQDFYDISVIDGFNLAMDF---
HvTLP2     SGSCQTGDCG-GQLSCS-LSGRPPATLAEFTIG--GGSTQDFYDISVIDGFNLAMDF---
HvTLP4     SGRCGTGDCG-GALSCT-LSGQPPLTLAEFTLG--GGT--DFYDISVIDGYNLPMDF---
HvTLP10    RGSCATGDCG-GVLACA-AGGKPPATLAEYTLG--TGGSPDFYDISLVDGFNVPMSFRP-
HvTLP5     RGRCITGDCA-GALVCR-VSGEQPATLAEYTLG--QGGNRDFFDLSVIDGFNVPMSFQP-
```

```
HvTLP6          RGRCITGDCN-GVLACR-VSGQQPTTLAEYTLG--QGANKDFFDLSVIDGFNVPMSFEP-
HvTLP7          RGRCITGDCG-GALACR-VSGQQPTTLAEYTLG--QGANKDFFDLSVIDGFNVPMSFEP-
HvTLP8          NGRCQTGDCG-GKLRCT-QYGQAPNTLAEFGLN--KYMGQDFFDISLIDGYNVPMSFVP-
HvTLP14         NGTCQTGDCQ-GMLACQ-TDGQPPYTSADFSLD--QYVNYSFFDISLYQGFNVPMEFLPM
HvTLP17         TGTCQTGDCG-GLLACK-ANGRPPNTIAEFALG--RPSNRDFFDISFVEGFNVPMDFLPE
                   * *.**  .   *       * *  :             :. :  .*:*: : .

HvTLP3          ---------------TCSSGDALRCREE--------------------------------
HvTLP13         -----HEGRGNCPVLACRKDLTQTCPGELQVRQGGGA----VVACRSGCLALGS---DEL
HvTLP16         ---RPANRSGGCFIAGCAKDMNAVCPPELQVTSGKKA---TVVACRSACLAFGL---DAF
HvTLP11         -------SHEGCQAAACAGDVNAACPSDLRVLSGGEV-----VACKSACNAYGS---ARY
HvTLP12         ---DNNGDGGACRAAACAGDVNAVCPSDLRVVSGSGE----VVACKSACNAYGS---ARY
HvTLP15         --TGTRVNSSTCGYAGCVGDVNALCPAELQVAGKEGAQEGKTVACRSACEAFGA---AEY
HvTLP18         QQGAAAAAGGNCAPTGCVVDLNGACPGDLRVPSSTGG-----VACKSACEAFGT---AQY
HvTLP9          -------SAPGCSNTGCLVDLNERCPSELRSGGG--------LACRSACEAFGK---PEF
HvTLP19         --AGSSATGTTCAAAGCSADLNLRCPAELRSAGG--------GACRSACDAFGK---PEY
HvTLP1          ---------------SCSTGDALQCRD---------------------------------
HvTLP2          ---------------SCSTGDALQCRD---------------------------------
HvTLP4          ---------------SCSTGVNIQCRD---------------------------------
HvTLP10         -------VAGSCHAISCAADINAKCPPELRVEGG----------CLSACVKLGV---PRY
HvTLP5          ------VGGAPCRAATCAVDITHECLPELQVPGG----------CASACGKFGG---DTY
HvTLP6          --------VGGCRAARCATDITKDCLKELQVPGG----------CASACGKFGG---DTY
HvTLP7          -------VGASCRAARCATDITKECLKELQVPGG----------CASACGKFGG---DTY
HvTLP8          ---APGSPGCPKGGPRCPKVITPACPNELRAAGG----------CNNACTVFKE---DRY
HvTLP14         PDKAQGGPGCTKGP-HCGASITSQCPSELKAPGG----------CNSACTVFQKQNYDMY
HvTLP17         P--AKGGPGCSKGP-RCGANITVQCPTSLKAPGG----------CNSPCTVFRQ---DRY
                                *       *

HvTLP3          -----------------------GCHDAFPYVEFNE----HACTAAGSRLQIVFCP----
HvTLP13         CCRNAYNSPAACRPSKYSDFFKSACPQAFTYAHDSPSLT-HECSAP-RELKVIFCH----
HvTLP16         CCRGAYGTAETCRGSVYSRIFRDACPAYYSYAYDVAAAT-PRCYA--QEYVLTFCPSRW-
HvTLP11         CCSGDYGTPAACGPTSYSQAFKAACPAAYSYAYDDASST-FTCSGA-DTYHITFCPAT--
HvTLP12         CCTGDYGTPTACGPTNYSRVFKTACPSAYSYAYDDASST-FTCAGA-TTYHITFCPIT--
HvTLP15         CCTGAHGGPDSCGPTKYSTLFKAACPAAYSYAYDDPTST-FTCGAG-AQYLITFCPAQ--
HvTLP18         CCSGEYGSPGTCRPSAYSQFFKNACPRAYSYAYDDATST-FTCAGAVSAYAITFCPSTT-
HvTLP9          CCNGAYGNPDTCHPSQYSQLFKSACPKSYSYAYDDATST-FTCNH--TDYTITFCPQSN-
HvTLP19         CCSGAFANPNTCHPTAYSQAFKLACPRSYSYAFDDPTST-FTCAGG-RDYTITFCPVAT-
HvTLP1          ---------PS-CPP-----------PQAYQHPNDVAT---HACSGN-NNYQITFCP----
HvTLP2          ---------PS-CPP-----------PQAYQHPNDVAT---HACSGN-NNYQITFCP----
HvTLP4          ----------------------RNCPDAYHTPPEPKT---KACSGN-RRFNIVFCP----
HvTLP10         CCTPPL-TPSTCGPTDYSRFFKGLCPDAYSYAYDDKSST-FTCPVG-SDYQLVFCP----
HvTLP5          CCRGQF--EHNCPPTYYSRFFKGKCPDAYSYAKDDQTST-FTCPAG-TNYQIVLCPARN-
HvTLP6          CCRGQF--EHNCPPTNYSMFFKGKCPDAYSYAKDDQTST-FTCPAG-TNYQIVLCP----
HvTLP7          CCRGQF--EHNCPPTNYSKFFKGKCPDAYSYAKDDQTST-FTCPAG-TNYQIVLCP----
HvTLP8          CCTGSA--ANSCGPTDYSRFFKGQCSDAYSYPKDDATSI-FTCPGG-TNYQVIFCP----
HvTLP14         CCE-----EPPCESNKYSAFFVRMCPEAISYSSDAPTKTSFSCRFN-TNYQVTFCPPLN-
HvTLP17         CCTGSA--ANNCSTTDYSGYFKKMCPDAYSYPRDDATSI-FSCPAG-TNYKVVFCPLTNQ
                                                          *        : :*

HvTLP3          ------------------------------------------------------------
HvTLP13         ------------------------------------------------------------
HvTLP16         -------GDRVAQA----------------------------------------------
HvTLP11         ------------------------------------------------------------
HvTLP12         ------------------------------------------------------------
HvTLP15         ------------------------------------------------------------
HvTLP18         -------SVKSAGSNAEAPAGLPL------------------------------------
HvTLP9          -------PYRYLPLNSF-------------------------------------------
```

```
HvTLP19         -------PSLKSAGGTTVVPTPTMPGSTGFAPPVMPRQAGGQADG---------------
HvTLP1          ------------------------------------------------------------
HvTLP2          ------------------------------------------------------------
HvTLP4          ------------------------------------------------------------
HvTLP10         ------------------------------------------------------------
HvTLP5          -------DLHMDQ-----------------------------------------------
HvTLP6          ------------------------------------------------------------
HvTLP7          ------------------------------------------------------------
HvTLP8          ------------------------------------------------------------
HvTLP14         -------LTSSPPSPPKSADLRTLRKRSSVGTSISVAIAGAIVSIVAFIFFIIRGRRTQR
HvTLP17         AVPPSPLPESPAGGTPVGPTTMKPRSSTARTVAAILAPVGGFIFLFIVAFYLCKRRIQRR

HvTLP3          ------------------------------------------------------------
HvTLP13         ------------------------------------------------------------
HvTLP16         ------------------------------------------------------------
HvTLP11         ------------------------------------------------------------
HvTLP12         ------------------------------------------------------------
HvTLP15         ------------------------------------------------------------
HvTLP18         ------------------------------------------------------------
HvTLP9          ------------------------------------------------------------
HvTLP19         ------------------------------------------------------------
HvTLP1          ------------------------------------------------------------
HvTLP2          ------------------------------------------------------------
HvTLP4          ------------------------------------------------------------
HvTLP10         ------------------------------------------------------------
HvTLP5          ------------------------------------------------------------
HvTLP6          ------------------------------------------------------------
HvTLP7          ------------------------------------------------------------
HvTLP8          ------------------------------------------------------------
HvTLP14         RQEMEEEEEEFGQLQGTPMRFTFQQLEAATEQFKDKLGEGGFGSVFEGQLGEERIAVKRL
HvTLP17         RETDEEEEEEFGELQGTPVRFTFEQLRAATEQFADKLGEGGFGSVFKGQFGNERIAVKRL

HvTLP3          ------------------------------------------------------------
HvTLP13         ------------------------------------------------------------
HvTLP16         ------------------------------------------------------------
HvTLP11         ------------------------------------------------------------
HvTLP12         ------------------------------------------------------------
HvTLP15         ------------------------------------------------------------
HvTLP18         ------------------------------------------------------------
HvTLP9          ------------------------------------------------------------
HvTLP19         ------------------------------------------------------------
HvTLP1          ------------------------------------------------------------
HvTLP2          ------------------------------------------------------------
HvTLP4          ------------------------------------------------------------
HvTLP10         ------------------------------------------------------------
HvTLP5          ------------------------------------------------------------
HvTLP6          ------------------------------------------------------------
HvTLP7          ------------------------------------------------------------
HvTLP8          ------------------------------------------------------------
HvTLP14         DRAGQGKREFLAEVQTIGSIHHINLVRLFGFCAEKSHRLLVYEYMSKGSLDKWIYARHEN
HvTLP17         DRTGQGKREFSAEVQTIGSIHHINLVRLIGFCAEKSHRLLVYEYMPKGSLDRWIYCRHED

HvTLP3          ------------------------------------------------------------
HvTLP13         ------------------------------------------------------------
```

```
HvTLP16          ------------------------------------------------------------
HvTLP11          ------------------------------------------------------------
HvTLP12          ------------------------------------------------------------
HvTLP15          ------------------------------------------------------------
HvTLP18          ------------------------------------------------------------
HvTLP9           ------------------------------------------------------------
HvTLP19          ------------------------------------------------------------
HvTLP1           ------------------------------------------------------------
HvTLP2           ------------------------------------------------------------
HvTLP4           ------------------------------------------------------------
HvTLP10          ------------------------------------------------------------
HvTLP5           ------------------------------------------------------------
HvTLP6           ------------------------------------------------------------
HvTLP7           ------------------------------------------------------------
HvTLP8           ------------------------------------------------------------
HvTLP14          SAPPLEWRVRCKVITDIAKGLSYLHEDCMKRIAHLDVKPQNILLDDDFNAKLSDFGLCKL
HvTLP17          DAPPLGWNTRCKIITHIAKGLSYLHEECTKRIAHLDVKPQNILLDDDFNAKLSDFGLCKL

HvTLP3           ------------------------------------------------------------
HvTLP13          ------------------------------------------------------------
HvTLP16          ------------------------------------------------------------
HvTLP11          ------------------------------------------------------------
HvTLP12          ------------------------------------------------------------
HvTLP15          ------------------------------------------------------------
HvTLP18          ------------------------------------------------------------
HvTLP9           ------------------------------------------------------------
HvTLP19          ------------------------------------------------------------
HvTLP1           ------------------------------------------------------------
HvTLP2           ------------------------------------------------------------
HvTLP4           ------------------------------------------------------------
HvTLP10          ------------------------------------------------------------
HvTLP5           ------------------------------------------------------------
HvTLP6           ------------------------------------------------------------
HvTLP7           ------------------------------------------------------------
HvTLP8           ------------------------------------------------------------
HvTLP14          IDRDMSQVVTRMRGTPGYLAPEWLTSQITEKADVYSFGVVVMEIVSGRKNLDTSLSEESI
HvTLP17          IDRDMSQVVTRMRGTPGYLAPEWLTSQITEKADIYSFGVVVMEIISGRKNLDTSRSEESI

HvTLP3           ------------------------------------------------------------
HvTLP13          ------------------------------------------------------------
HvTLP16          ------------------------------------------------------------
HvTLP11          ------------------------------------------------------------
HvTLP12          ------------------------------------------------------------
HvTLP15          ------------------------------------------------------------
HvTLP18          ------------------------------------------------------------
HvTLP9           ------------------------------------------------------------
HvTLP19          ------------------------------------------------------------
HvTLP1           ------------------------------------------------------------
HvTLP2           ------------------------------------------------------------
HvTLP4           ------------------------------------------------------------
HvTLP10          ------------------------------------------------------------
HvTLP5           ------------------------------------------------------------
HvTLP6           ------------------------------------------------------------
HvTLP7           ------------------------------------------------------------
HvTLP8           ------------------------------------------------------------
```

```
HvTLP14         HLITLLEEKVKSDHLEDLIDKSSNNMQADKRDAIQMMKLAMWCLQIDCKKRPKMSEVVKV
HvTLP17         HLITLLEEKVKSDRLVDLIDNNSNDMQAHKQDVIQMMMLAMWCLQIDCKKRPKMFEVVKV


HvTLP3          ---------------------------------------------
HvTLP13         ---------------------------------------------
HvTLP16         ---------------------------------------------
HvTLP11         ---------------------------------------------
HvTLP12         ---------------------------------------------
HvTLP15         ---------------------------------------------
HvTLP18         INGTMVYQGGDQFVGAGAAARPALDLAAVLVGVAALALARPMPR
HvTLP9          ---------------------------------------------
HvTLP19         QGVILGDNSWLANMATGDMSAATPSRTAMIPAAPLALLILRLLL
HvTLP1          ---------------------------------------------
HvTLP2          ---------------------------------------------
HvTLP4          ---------------------------------------------
HvTLP10         ---------------------------------------------
HvTLP5          ---------------------------------------------
HvTLP6          ---------------------------------------------
HvTLP7          ---------------------------------------------
HvTLP8          ---------------------------------------------
HvTLP14         LEGTMDADSNIDHNFVATNEANFGIAGNANSSAPPIATDLSGPR
HvTLP17         LDGTMTADLNIEHNFVVTTSANFRSTGNVSSSDPPLASDVSGPR
```

Figure S2. Amino acid sequence alignment of barley HvTLPs. The highlighted sequences indicate the novel TLP11-14, TLP16, TLP17 and TLP19 having >16 cysteine residues. Sequence alignment was performed by using the MUSCLE alignment tool.
