# Supplementary material for: Thaumatin-Like Protein (TLP) Gene Family in Barley: Genome-Wide Exploration and Expression Analysis during Germination
Source: Genes (Basel). 2020 Sep 16;11(9):1080. doi: 10.3390/genes11091080 (PMC7564728; doi:10.3390/genes11091080)
Supplement: Supplementary file 1 [file genes-11-01080-s001.zip › Figure S3.pdf]

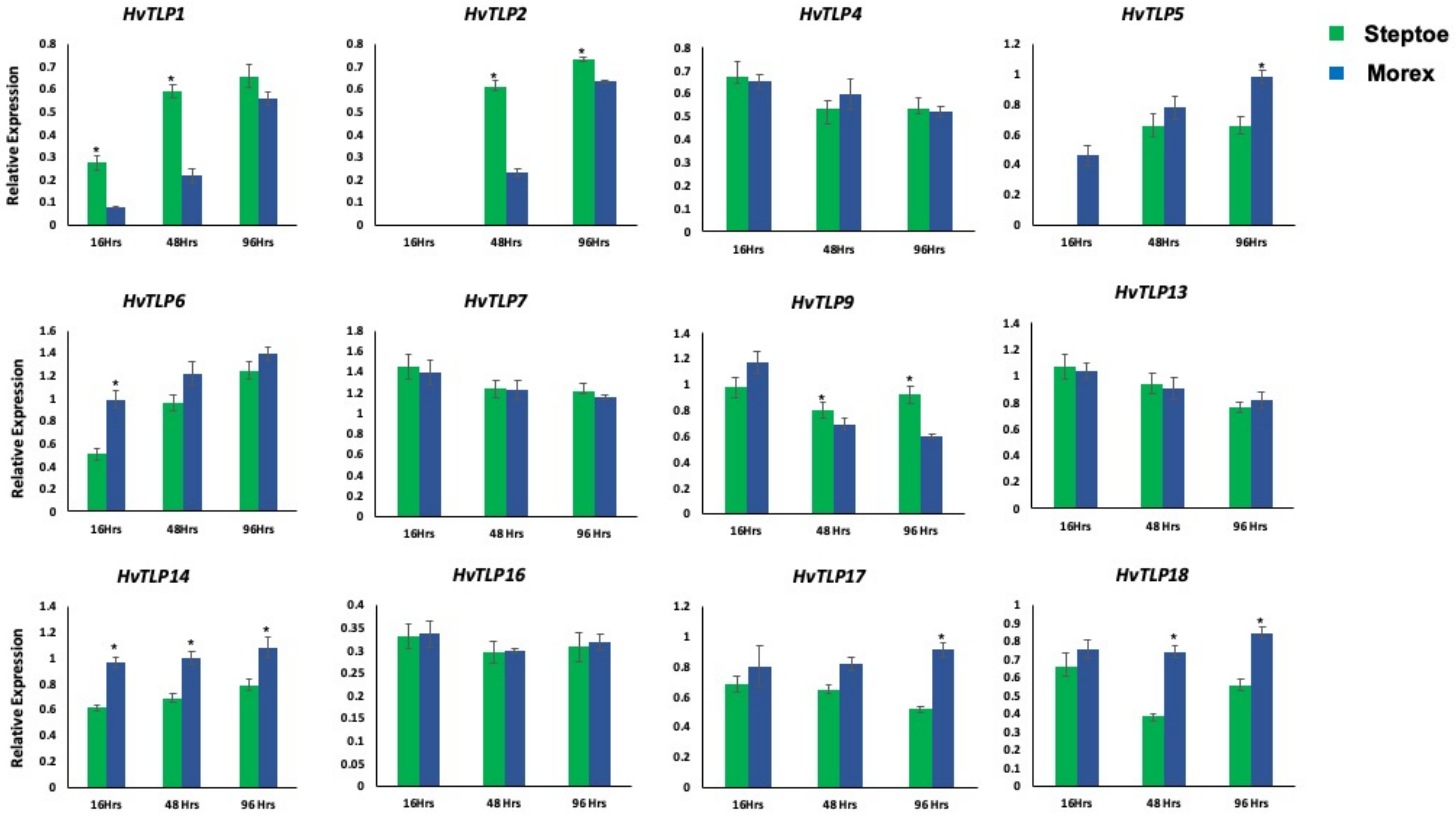

Figure S3. Transcript levels of *HvTLPs* in malt (Morex) and feed (Steptoe) varieties during different stages of seed germination. Error bars were obtained from two measurements. Asterisk (*) above the bars indicates significant ($p < 0.05$) differences of expression levels between malt and feed varieties.
