# Supplementary figures and images for: Thaumatin-Like Protein (TLP) Gene Family in Barley: Genome-Wide Exploration and Expression Analysis during Germination
Source: Genes (Basel). 2020 Sep 16;11(9):1080. doi: 10.3390/genes11091080 (PMC7564728; doi:10.3390/genes11091080)

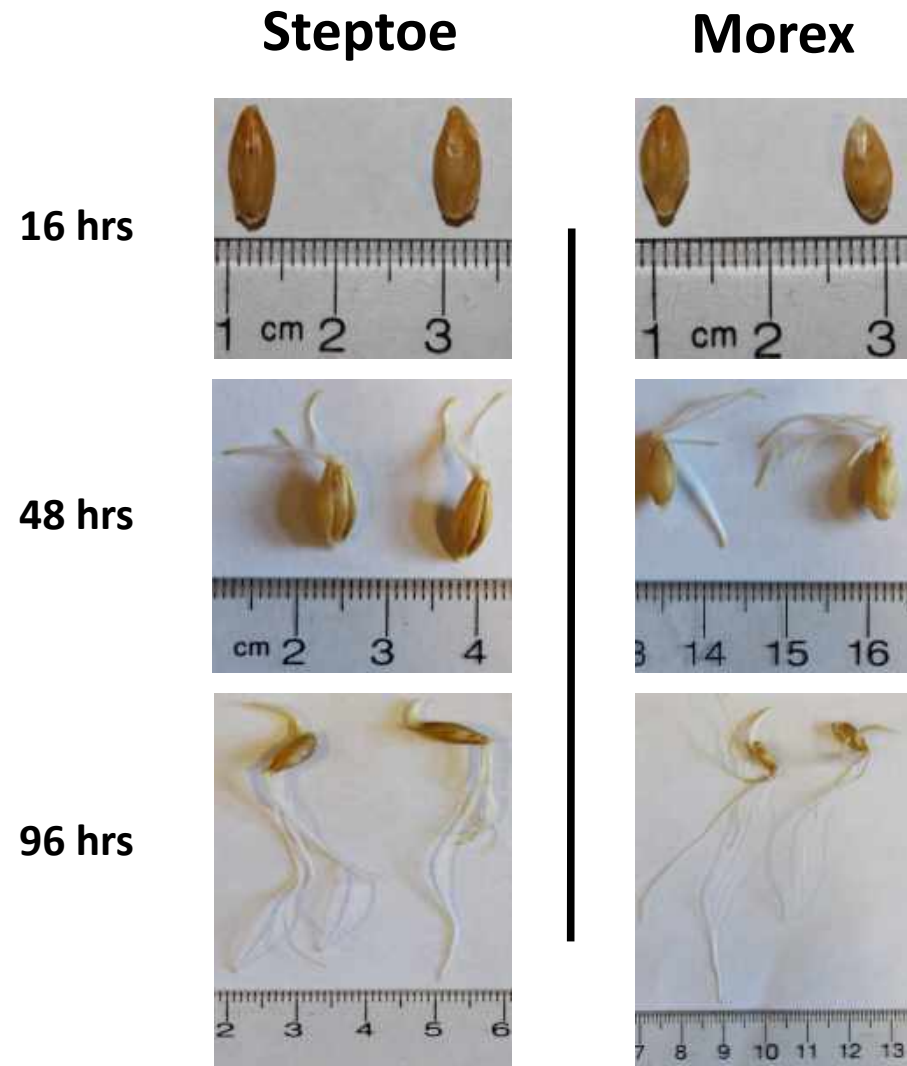

**Figure S1.** Grain germination in Steptoe and Morex at different growth stages.

Supplement: Supplementary file 1 [file genes-11-01080-s001.zip › Figure S1.pdf]
